# Safety Studies for Use of Adipose Tissue‐Derived Mesenchymal Stromal/Stem Cells in a Rabbit Model for Osteoarthritis to Support a Phase I Clinical Trial

**DOI:** 10.5966/sctm.2016-0097

**Published:** 2016-10-26

**Authors:** Scott M. Riester, Janet M. Denbeigh, Yang Lin, Dakota L. Jones, Tristan de Mooij, Eric A. Lewallen, Hai Nie, Christopher R. Paradise, Darcie J. Radel, Amel Dudakovic, Emily T. Camilleri, Dirk R. Larson, Wenchun Qu, Aaron J. Krych, Matthew A. Frick, Hee‐Jeong Im, Allan B. Dietz, Jay Smith, Andre J. van Wijnen

**Affiliations:** ^1^ Department of Orthopedic Surgery, Mayo Clinic College of Medicine, Mayo Clinic, Rochester, Minnesota, USA; ^2^ Department of Orthopedic Surgery, Tongji Hospital, Tongji Medical College, Huazhong University of Science and Technology, Wuhan, People’s Republic of China; ^3^ Department of Biomedical Engineering and Physiology, Mayo Graduate School, Mayo Clinic, Rochester, Minnesota, USA; ^4^ Department of Laboratory Medicine and Pathology, Mayo Clinic College of Medicine, Mayo Clinic, Rochester, Minnesota, USA; ^5^ Department of Health Sciences Research, Mayo Clinic College of Medicine, Mayo Clinic, Rochester, Minnesota, USA; ^6^ Department of Physical Medicine and Rehabilitation, Mayo Clinic College of Medicine, Mayo Clinic, Rochester, Minnesota, USA; ^7^ Department of Anesthesiology, Division of Pain Medicine, Mayo Clinic College of Medicine, Mayo Clinic, Rochester, Minnesota, USA; ^8^ Department of Radiology, Mayo Clinic College of Medicine, Mayo Clinic, Rochester, Minnesota, USA; ^9^ Department of Biochemistry, Rush University Medical Center, Chicago, Illinois, USA; ^10^ Department of Orthopedic Surgery, Rush University Medical Center, Chicago, Illinois, USA; ^11^ Section of Rheumatology, Department of Internal Medicine, Rush University Medical Center, Chicago, Illinois, USA; ^12^ Jesse Brown Veterans Affairs Medical Center, Chicago, Illinois, USA; ^13^ Department of Anatomy, Mayo Clinic College of Medicine, Mayo Clinic, Rochester, Minnesota, USA; ^14^ Department of Biochemistry and Molecular Biology, Mayo Clinic College of Medicine, Mayo Clinic, Rochester, Minnesota, USA

**Keywords:** Cell therapy, Stem cell, Transplant, Regenerative medicine, Osteoarthritis, Rabbit

## Abstract

Adipose‐derived mesenchymal stem cells (AMSCs) offer potential as a therapeutic option for clinical applications in musculoskeletal regenerative medicine because of their immunomodulatory functions and capacity for trilineage differentiation. In preparation for a phase I clinical trial using AMSCs to treat patients with osteoarthritis, we carried out preclinical studies to assess the safety of human AMSCs within the intra‐articular joint space. Culture‐expanded human AMSCs grown in human platelet‐lysate were delivered via intra‐articular injections into normal healthy rabbit knees and knees at risk for the development of osteoarthritis after bilateral medial anterior hemimeniscectomy. Treatment outcomes and safety were evaluated by assessing the general health, function, and behavior of the animals. Joint tissues were analyzed by x‐ray, magnetic resonance imaging, and histopathology. Intra‐articular AMSC therapy was well tolerated in this study. We did not observe adverse systemic reactions, nor did we find evidence of damage to intra‐articular joint tissues. Thus, the data generated in this study show a favorable safety profile for AMSCs within the joint space in support of a phase I clinical trial evaluating the clinical utility of AMSCs to treat osteoarthritis. Stem Cells Translational Medicine *2017;6:910–922*

**Significance Statement**

This safety study indicates that adipose‐derived mesenchymal stem cells (AMSCs) can be safely used in cell therapies for joint degeneration. This finding supports a phase I clinical trial to evaluate the clinical utility of AMSCs in treating osteoarthritis.

## Introduction

Osteoarthritis (OA) is a major musculoskeletal degenerative disease that causes significant physical disability and pain. It is estimated that approximately 7 million Americans are currently living with a hip or knee replacement secondary to osteoarthritis‐related joint degeneration [1]. By 2030, in the U.S. alone, there are projected to be almost 4 million primary total knee or hip arthroplasties performed annually for osteoarthritis‐related joint degeneration [2]. Articular cartilage has a reduced innate regenerative capacity [3]; however, a number of clinical therapies are commonly used for OA treatment. These include oral nonsteroidal anti‐inflammatory medications, viscosupplementation (i.e., SYNVISC and Supartz), platelet‐rich plasma, and intra‐articular corticosteroid therapies [4, 5, 6, 7]. Intra‐articular corticosteroid injections have been used for decades to treat osteoarthritis pain symptoms, and they currently serve as the primary treatment alternative to joint replacement for end‐stage OA. Although effective at relieving acute pain symptoms, their use over time may contribute to cartilage breakdown and accelerated progression of osteoarthritis [8]. These current treatments are primarily aimed at symptomatic relief of pain symptoms and are associated with significant potential for adverse side effects. To date, there are no therapies in clinical use that have been shown to clearly slow or reverse osteoarthritis progression.

Adipose‐tissue derived mesenchymal stromal/stem cells (AMSCs) are immature mesenchymal cells with stem cell‐like properties that are derived from the stromal vascular fraction of adipose tissue. AMSCs are the focus of our studies because they can be more easily obtained—and in vastly greater quantities—than mesenchymal stromal cells (MSCs) from other sources. As such, AMSCs are amenable to therapeutic applications that require or would benefit from autologous cell transplants. Furthermore, AMSCs expanded in human platelet lysate are zoonotic‐free cells that have promising potential for clinical orthopedic applications, based on their ability to differentiate into different types of mesenchymal cells, including those of the osteogenic, chondrogenic, and adipogenic lineages [9, 10, 11, 12]. In addition to their capacity for multilineage differentiation, these cells may support tissue regeneration via their trophic and secretory functions, including anti‐inflammatory activity [13, 14]. During OA pathogenesis, joint inflammation promotes a catabolic state characterized by the loss of extracellular matrix, in which chondrocytes are susceptible to cell death and apoptosis [15]. The anti‐inflammatory activity of AMSCs may relieve pain symptoms of OA without the risk of cartilage degeneration that can occur with corticosteroid use.

In this study, we address the important question of whether platelet‐lysate expanded AMSCs can be safely used within the intra‐articular joint space. We examine the biological effects of injecting human AMSCs into the intra‐articular knee space of normal healthy knees (cohort A) and of knees at risk for the development of osteoarthritis secondary to medial anterior hemimeniscectomy (cohort B) using a translational rabbit model for OA. The main finding of our study is that AMSCs are well‐tolerated in both models, even though we observed minor inflammatory responses that are attributable to cell xenografts in an immunocompetent animal. This study provides preliminary safety data in support of an approved investigational new drug application to the Food and Drug Administration for initiation of a phase I clinical trial for in human use of autologous AMSCs to treat osteoarthritis.

## Materials and Methods

### Overview of the Study Design

**Figure 1:**
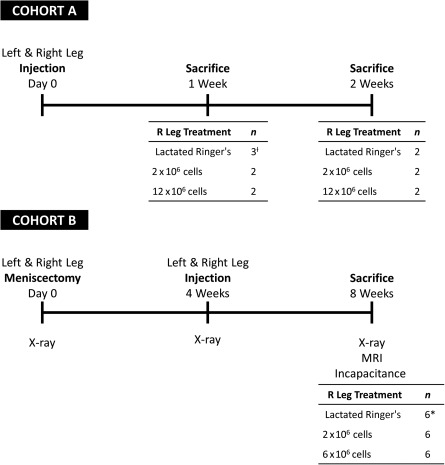
Study design. An experimental timeline showing the procedures for cohorts A and B. Top: Cohort A: Injection of right and left rabbit knees at time zero (left) followed by sacrifice and tissue harvest at either 7 days after injection (center) or 14 days after injection (right). The left knee of each rabbit was injected with lactated Ringer’s while the right knees were treated with either lactated Ringer’s, 2 × 10^6^ cells, or 12 × 10^6^ cells. Bottom: Cohort B: Injection of right and left rabbit knees at 4 weeks after bilateral anterior hemimeniscectomies. The left knee of each rabbit was injected with lactated Ringer’s, while the right knees were treated with lactated Ringer’s, 2 × 10^6^ cells, or 6 × 10^6^ cells. Animals in this cohort were sacrificed 4 weeks after injection (8 weeks after partial meniscectomy). ƚ, One rabbit in cohort A receiving lactated Ringer’s injections in both knees developed knee swelling and systemic illness consistent with septic arthritis and was euthanized 4 days after injection. ∗, A rabbit scheduled to receive a lactated Ringer’s injection into its right knee was sacrificed before receiving injection therapy because of postoperative complications related to the surgical meniscectomy procedure. Abbreviation: R, right.

The safety profile of human AMSCs was evaluated in two cohorts of mature female New Zealand white rabbits (12 months old, body weight 2.5–3.5 kg) (Harlan Laboratories/Envigo, Oxford, MI, http://www.envigo.com) In the first group (cohort A, *n* = 13), healthy rabbits received intra‐articular knee injections of AMSCs at a dose of either 2 × 10^6^ or 12 × 10^6^ cells or lactated Ringer’s control into their right knee. Investigators performing the injections and evaluating the rabbit vitals and tissue histology were blinded to the type of experimental treatment being administered. Additionally, 1 ml of lactated Ringer’s control solution was also injected into the left knee of each rabbit as an internal control. Rabbits were sacrificed at either 7 days (2 × 10^6^ cells: *n* = 2; 12 × 10^6^ cells: *n* = 2; lactated Ringer’s control: *n* = 3) or 14 days (2 × 10^6^ cells: *n* = 2; 12 × 10^6^ cells: *n* = 2; lactated Ringer’s control: *n* = 2) after experimental injection (Fig. 1). Safety and treatment outcomes were evaluated by daily measurement of rectal temperature and body weight, as well as visual inspection of the injection site and knee joint. Joint tissues were fixed and histologically evaluated at the completion of the study.

Because our ultimate goal is to use AMSCs to treat OA in patients, we also examined the safety of AMSCs in a second group of rabbits at risk for osteoarthritis development secondary to partial medial anterior hemimeniscectomy. These rabbits (cohort B; *n* = 18) underwent bilateral medial anterior hemimeniscectomies, followed by experimental injections of either AMSCs at a dose of 2 × 10^6^ cells (*n* = 6) or 6 × 10^6^ cells (*n* = 6) or lactated Ringer’s control (*n* = 6) into their right knee 4 weeks later (Fig. 1). Treatment outcomes and safety were evaluated by assessing weekly rectal temperature and body weight. The rabbit’s surgical incision and injection sites were also visually inspected daily. Additionally, the effect of AMSC therapy on intra‐articular joint structures was assessed by using x‐ray, magnetic resonance (MR) imaging, and histologic evaluation of joint tissues. Before animal sacrifice, a pain evaluation was performed by using weight‐bearing measures (incapacitance testing). All animal studies were carried out with approval from the Mayo Clinic's Institutional Animal Care and Use Committee.

### Primary Culture of Human AMSCs

Human AMSCs were cultured from adipose tissue collected from patients undergoing elective liposuction surgery in compliance with protocols approved by the Mayo Clinic's institutional review board. For specific details about human AMSC harvesting, and in vitro culturing, refer to Crespo‐Diaz et al. [11]. In brief, AMSCs were cultured in advanced minimum essential medium (Thermo Fisher Scientific Life Sciences, Oakwood Village, OH, https://www.thermofisher.com) supplemented with 5% (vol/vol) human platelet lysate (PLTMax, Mill Creek Life Sciences, Rochester, MN, http://www.millcreekls.com), 1% (vol/vol) Penn‐Strep (100 U/ml penicillin and 100 μg/ml streptomycin; Corning, Corning, NY, http://www.corning.com), 2 mM Glutamax (Thermo Fisher), and 2 U/ml heparin (Vizient, Irving, TX, https://www.vizientinc.com). Standard operating procedures were followed according to good manufacturing practice guidelines. Passage 5 cells were used for the injection procedures to ensure that a sufficient number of cells would be available for both molecular testing and animal injection studies. Additionally, AMSCs have been shown to maintain their proliferative potential at later passages when grown in human platelet lysate, compared with adult‐derived stem cells grown in fetal bovine serum [11]. Stem cell populations were confirmed by using established criteria from the International Society for Cellular Therapy and the International Federation for Adipose Therapeutics and Science [16]. All cells were characterized according to their cell surface profile and were positive for CD44, CD73, CD90, CD105, and HLA‐ABC and negative for CD14, HLA‐DR, and CD45. These cell surface markers have previously been validated as markers that define an adipose‐derived stem cell population with trilineage potential [9, 10, 11]. At the time of cell preparation, AMSCs were detached from flasks by using TrypLE Express (Thermo Fisher) and counted by using a hemocytometer (Thermo Fisher). Cells were suspended in 1 ml of lactated Ringer’s solution at concentrations of (a) 2 × 10^6^ or (b) 12 × 10^6^ cells per milliliter for cohort A and at concentrations of (c) 2 × 10^6^ or (d) 6 × 10^6^ cells per milliliter for cohort B. Cell suspensions were stored on wet ice until ready for administration. The higher cell dosing regimen was used in cohort A to evaluate for potential acute adverse reactions, including systemic and localized immune reactions. For cohort B, we used lesser doses to simulate clinically relevant scenarios. We used doses that were similar to those used in previous studies when accounting for scaling differences between human and rabbit knees [17], because some studies have suggested that high‐dose therapy (doses greater than 150 million cells injected into a human knee) may be less efficacious [18].

### Rabbit Meniscectomy

**Figure 2:**
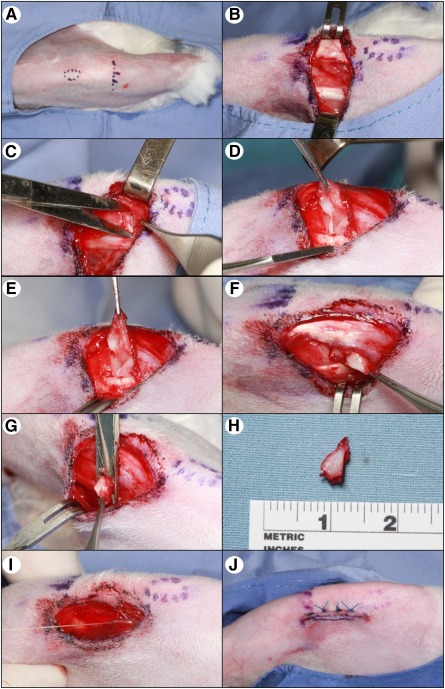
Rabbit meniscectomy. Surgical procedure for medial anterior hemimeniscectomy. **(A):** Rabbit knee after shaving and anatomical marking. **(B):** A vertical incision made along the medial aspect of the knee joint reveals the patellar tendon and medial collateral ligament. **(C):** An incision is made along the joint line to expose the meniscus. **(D):** The infrapatella fat pad is retracted, exposing the lateral insertion of the medial meniscus. **(E):** The lateral insertion site is released. **(F):** The meniscus is reflected medially out of the joint. **(G):** Its attachment at the medial collateral ligament is released. **(H):** The resected medial meniscus. **(I):** The fascia and skin are then closed with sutures. **(J):** The rabbit knee after surgery.

To increase the susceptibility of the rabbit knees to osteoarthritis development, we performed bilateral medial anterior hemimeniscectomies on all rabbits in cohort B, before AMSC injection (Fig. 2). The development of OA is generally characterized by macroscopically visible surface fibrillation and focal erosions/lesions that increase in number and severity to 12 weeks and beyond [19, 20, 21, 22, 23], as well as osteophyte development [24].

Anesthesia was carried out via administration of an intramuscular injection of ketamine (35–75 mg/kg) and 5 mg of xylazine, with inhalation of isoflurane (1%–4%). Rabbits were intubated with a 2.5–3.5 endotracheal tube and maintained on 100% oxygen and isoflurane for the procedure. Buprenorphine SR 0.18 mg/kg (subcutaneous; s.c.) was given for pre‐emptive analgesia, as well as cefazolin (22 mg/kg) i.v. to prevent infections. Body temperature was monitored by using a rectal thermometer, with a recirculating warm water pad used as necessary. Once anesthetized, electric clippers were used to remove the hair overlying each knee, and the skin was prepared by using Hibiclens (Mölnlycke Health Care U.S., Norcross, GA, http://www.molnlycke.us). Sterile techniques were used during the operation. A paramedian vertical skin incision (1–2 cm) was made parallel to the patellar tendon, and an arthrotomy was performed. The anterior insertional ligament of the medial meniscus was released, and the medial aspect of the meniscus was dissected free from its attachment at the medial collateral ligament. The joint was irrigated with normal saline, and the wound was closed in layers, using 3‐0 Vicryl interrupted simple sutures for the fascia and the ethylon 5‐0 interrupted horizontal mattress suture for the skin (Fig. 2). Carprofen 4 mg/kg s.c. was given at the time of surgery and once every 24 hours for 3 days postoperatively for pain control.

After surgery, rabbits were placed back into a holding pen, where they were allowed to recover; they were not transported until they had regained mobility. Every animal was physically examined twice within the first 24 hours. One rabbit in cohort B was euthanized because of an infection of the left knee after meniscectomy, before the injection of AMSCs. All other animals in this cohort recovered normally and underwent a single bilateral intra‐articular knee injection of human AMSCs 4 weeks after the meniscectomy.

### Intra‐Articular Rabbit Knee Injections

**Figure 3:**
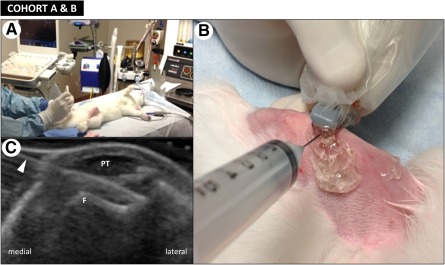
Injection of AMSCs into anesthetized rabbits. Intra‐articular knee injection procedure after shaving, with the injection of stem cells into the rabbit knee joint under ultrasound image guidance, is shown. **(A):** The rabbit is anesthetized and positioned in the supine position on the operating table. **(B):** A 19‐gauge needle is inserted into the intra‐articular space along the joint line under ultrasound guidance. **(C):** Ultrasound imaging shows the needle (arrowhead) passing between the patellar tendon and the femur positioned within the trochlear groove. Abbreviations: F, femur; PT, patellar tendon.

Before intra‐articular injection of either AMSCs or lactated Ringer’s treatment, each rabbit was anesthetized with an intramuscular injection of ketamine (35 mg/kg) and xylazine (5 mg/kg), intubated, and prepared as described above for the meniscectomy procedure. A 3‐ml plastic syringe (BD Biosciences, San Jose, CA, http://www.bdbiosciences.com) was loaded with 1 ml of lactated Ringer’s solution either with or without a cell suspension, to which a 19‐gauge stainless steel needle (Medtronic, Minneapolis, MN, http://www.medtronic.com) was subsequently attached. The needle was inserted into the knee joint, and the experimental therapy was delivered under ultrasound guidance (CX50 ultrasound machine, Linear 12‐3 MHz probe, Philips Healthcare, Fitchburg, WI, http://www.usa.philips.com) with the use of sterile ultrasound gel to ensure appropriate intra‐articular delivery. In both study cohorts, ultrasound imaging was used to identify anatomic landmarks within the knee joint (Fig. 3). The needle was inserted into the knee joint between the patellar tendon and trochlea by using a single pass, in plane approach to visualize the shaft and tip of the needle. Special care was taken to avoid iatrogenic cartilage injury during the injection. The injectate was observed to distend the suprapatellar recess, indicating successful delivery of the experimental therapy into the knee. After injection, the knee was placed through several ranges of motion and re‐examined with ultrasound (US) to confirm joint distention. At the completion of the procedure, each animal received 120 ml of PlasmaLyte (Baxter, Utrecht, The Netherlands, http://www.baxter.nl) s.c. for recovery; no antibiotics or analgesics were administered.

### Pain Evaluation (Incapacitance Testing)

To determine whether rabbits experienced measurable differences in discomfort or pain sensation between knees treated with AMSCs and lactated Ringer’s control, the weight distribution on the two hind limbs of rabbits in cohort B (at 8 weeks after meniscectomy) were measured by using an incapacitance meter (Incap, Columbus Instruments, Columbus, OH, http://www.colinst.com). Alert rabbits were measured in a neutral (semiupright; using the manufacturer‐provided animal restrainer) or elevated (forelimbs lifted while eyes covered) position with hind paws placed on separate sensor pads, and the force exerted by each limb (measured in grams) averaged over 5 seconds per reading. To minimize distress during testing, the eyes of each rabbit were covered. Incapacitance measurements for each rabbit were taken three times in both the neutral and semiupright positions.

### Animal Sacrifice

At the completion of the study, rabbits were euthanized either 1 or 2 weeks after injection (cohort A) to evaluate for acute adverse reactions and at 4 weeks after injection (cohort B) to evaluate for chronic adverse effects on cartilage tissues. Euthanization was carried out via intracardiac administration of 3 ml of Fatal Plus (392 mg/ml pentobarbital sodium) (Vortech Pharmaceuticals, Ltd., Dearborn, MI) after a ketamine (35 mg/kg) and xylazine (5 mg/kg) injection. Immediately after sacrifice, the animals in cohort B underwent x‐ray and MR imaging before tissue harvest.

### X‐Ray Imaging

The hind limbs of animals in cohort B were imaged immediately after surgery, at 4 weeks (after anesthetization), and at 8 weeks (immediately after sacrifice) postoperation by using anterior‐posterior (AP) and lateral x‐rays to look for signs of OA or other joint damage (e.g., osteophytes). X‐rays of both knees were taken by using the HF 100\30+ Ultra light (MinXray Inc., Northbrook, IL, http://www.minxray.com). X‐ray radiographs were then digitized (ScanX, ALLPRO Imaging, Melville, NY, http://allpro‐imaging.com). Radiographs were evaluated by a trained orthopedic surgeon, who was blinded to the treatment type administered in the right knee. X‐rays were evaluated for signs of OA and joint destruction through assessment of osteophyte formation, joint‐space narrowing, sclerosis, and joint deformity.

### Magnetic Resonance Imaging

Magnetic resonance imaging with T2‐weighting was performed on rabbit hind limbs in cohort B to assess potential pathological changes in the articular cartilage of the knees [25, 26]. After sacrifice and x‐ray imaging, both rabbit legs were disarticulated and prepared for MR scanning. The legs were bound in the fully extended position and placed between custom manufactured paired 2‐channel receive only phased array coils (Mayo Clinic MR Laboratory, Rochester MN, http://www.mayoclinic.org). Imaging was performed on a 3.0‐T MRI (Signa, GE Healthcare Life Sciences, Piscataway, NJ, http://www.gelifesciences.com). Fast‐spin echo axial fat‐saturated T2‐weighted images and matched sagittal proton density and fat‐saturated T2‐weighted images were obtained through both knees for each rabbit specimen using the following parameters: field of view = 8 × 8 cm; repetition time = 4,000 milliseconds; echo time = 40–60 milliseconds; matrix = 512 × 320 with 2‐mm slice thickness, no interslice gap, and number of excitations = 2. T2‐weighted maps were generated for each rabbit specimen by using an Advantage Windows workstation, platform 4.3 (GE Healthcare). The average T2 relaxation times for the articular cartilage corresponding to the medial and lateral femoral condyles and medial and lateral tibial plateau of both the left and right knees of each animal were measured within a region of interest (4 mm^2^).

### Tissue Histology

Joint tissues were harvested immediately after sacrifice in cohort A and after x‐ray and MR imaging in cohort B. The distal femur (cohort A only), proximal tibia, lateral meniscus, synovium, and fat pad were fixed in 10% neutral buffered formalin for 72 hours in preparation for paraffin embedding. The articular surface and associated subchondral bone from the proximal tibia and distal femur were sectioned into four quadrants and decalcified by using the RDO Rapid Decalcifying Solution (Apex Engineering Products Corp., Aurora, IL, http://www.apexengineeringproducts.com) for several hours until the bone tissue was completely decalcified. Tissue specimens were embedded in paraffin and cut into 5‐μm sections for staining. All specimens were mounted onto slides and stained using protocols for hematoxylin and eosin (H&E) staining. In addition, all cohort B tissues from rabbits receiving injections of either lactated Ringer’s or 6 × 10^6^ AMSCs into their right (experimental) knees were also stained for safranin O and counterstained with fast green so that Osteoarthritis Research Society International (OARSI) scores could be evaluated [26, 27]. Histological sections were digitized by using a stage scanner microscope (Mikroscan D2, Mikroscan Technologies, Inc., Carlsbad, CA, http://www.mikroscan.com). Histopathology of stained slides was independently evaluated by an experienced veterinary pathologist (Department of Veterinary Pathology, Mayo Clinic) who was blinded to the different treatment arms of the study. General pathological observations were provided for all H&E slides. Articular cartilage changes in the tissues of rabbits at risk of osteoarthritis (after bilateral medial anterior hemimeniscectomies) were evaluated according to the OARSI guidelines for evaluation of rabbit tissues [26, 27]. Samples in cohort B were scored for the following: staining quality of safranin O‐fast green (0–6), structure of cartilage (0–11), decreases in chondrocyte density (0–4), and cluster formation (0–3). A histopathological assessment of OA synoviopathy in the right and left synovium and fat pad tissues of cohort B animals receiving injections of either (a) lactated Ringer’s or (b) 6 × 10^6^ AMSCs in the right leg was also conducted according to the 0–3 scoring criteria for synoviocytes, inflammatory infiltrates, and synovial stroma parameters outlined by Laverty et al. [26].

### Statistical Analysis

Descriptive statistics were used to assess the effects of AMSC injection on cohort A and B animals. Mean values and SDs were reported unless otherwise indicated. A general linear model analysis was performed in SAS (Version 9.4, SAS Institute Inc., Cary, NC, http://www.sas.com) to test whether there were any significant differences between left and right leg incapacitance measurements. A repeated‐measures analysis of variance (ANOVA) was performed to investigate effects related to treatment type (lactated Ringer’s or AMSC) and/or leg. Reliability of the measurements was described by using intraclass correlations and coefficients of repeatability. Statistical comparisons of T2 mapping time (means) between left and right legs were assessed by using paired *t* tests. Significant differences in right leg mapping times across treatment type were also tested by using ANOVA. In all cases, statistical significance was defined as *p* < .05.

## Results

### Safety Evaluation of Human AMSCs After Injection Into Healthy Rabbit Knees

#### Evaluation of Systemic Adverse Reactions in Healthy Rabbits

For our initial safety evaluation of human AMSC therapy within the joint space, we performed intra‐articular injections of AMSCs or lactated Ringer’s control into healthy knee joints of mature New Zealand white rabbits (cohort A). After injection, weight, body temperature, injection site appearance, and behavior were recorded daily. One rabbit receiving lactated Ringer’s injections in both knees developed knee swelling and systemic illness consistent with septic arthritis. This rabbit was euthanized 4 days after injection. All other rabbits maintained normal behavior and stable vital signs. Excluding the rabbit that developed septic arthritis, animals were sacrificed at either 7 (*n* = 6) or 14 (*n* = 6) days after injection therapy so that intra‐articular tissues could be collected and assessed for acute adverse reactions (e.g., cartilage damage).

#### Assessment of Tissue Morphology/Histology in Healthy Rabbit Knees

**Figure 4:**
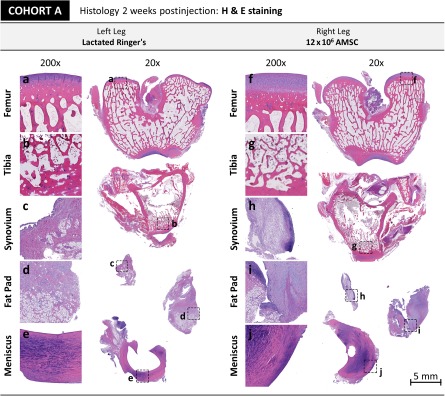
Cohort A histology. Representative H&E‐stained histological samples of cohort A rabbit right knees injected with 12 × 10^6^ AMSCs and the corresponding control left leg (lactated Ringer’s) are shown. Tissues include the femur, tibia, synovium, infrapatellar fat pad, and meniscus. **(a–e):** No significant pathology was observed in left leg tissues. **(f–j):** On the right side, mild to moderate aggregation of lymphoplasmacytic cells was observable in all tissues. No tissue injury/damage associated with the infiltrate was observed. Scale bar = 5 mm. Abbreviations: AMSC, adipose‐derived mesenchymal stem cell; H&E, hematoxylin and eosin staining.

Upon gross visual inspection, all knees appeared healthy with smooth articular cartilage. Right knees receiving experimental treatment (2 × 10^6^ or 12 × 10^6^ cells or lactated Ringer’s solution) appeared indistinguishable from the contralateral left control knees for all treatment groups (data not shown). Histologic evaluation of knee tissues including the tibia, meniscus, synovium, and fat pad, showed no evidence of cartilage injury or damage to other intra‐articular joint tissues at either the day 7 or 14 time points. At 7 days, the two rabbits receiving injections of 2 × 10^6^ AMSCs developed lymphoplasmacytic infiltrates in their right knee; one of these rabbits also developed infiltrates in their contralateral control knee. The two rabbits receiving a dose of 12 × 10^6^ AMSCs also developed lymphoplasmacytic infiltrates (Fig. 4). One of the rabbits developed infiltrates in both knees, whereas the other rabbit only developed lymphoplasmacytic infiltrates in the contralateral control knee. In addition, one of the rabbits receiving only lactated Ringer’s therapy developed a mild lymphoplasmacytic infiltrate in the left knee, whereas the other rabbit did not develop any lymphoplasmacytic infiltrates. These data show that early lymphoplasmacytic infiltrates can be observed in rabbit knees receiving either AMSC or lactated Ringer’s therapy. These cellular infiltrates could be attributable to inflammation caused by the injection procedure or the injectate itself, as well as associated joint distension.

At 14 days, all of the rabbits that received AMSC injections (at a dose of either 2 × 10^6^ or 12 × 10^6^ AMSCs) had developed lymphoplasmacytic infiltrates in their right knees, whereas their contralateral control knees were free of any cellular infiltrates. A single case of hyperplasia was present in the left synovium of one rabbit. All of the rabbits that had only received lactated Ringer’s therapy were also free of lymphoplasmacytic infiltrates. These data suggest a persistent subclinical lymphoplasmacytic cellular infiltration in rabbit joints receiving human AMSC therapy. This effect may be due to an immune response to the xenogeneic human cells. Representative histological pictures of control‐treated and AMSC‐treated knees from cohort A are presented in Figure 4.

### Safety Evaluation of AMSCs After Injection Into Rabbit Knees at Risk for Osteoarthritis Secondary to Partial Meniscectomy

Patients who are at risk for rapid osteoarthritis development, such as patients with meniscal injuries, are potential candidates for AMSC therapy because of its potential disease‐modifying effects. We therefore wanted to evaluate the safety of AMSCs within joints at risk for osteoarthritis development, to make sure they would not contribute to rapid disease progression in high‐risk joints. For these studies, we performed a bilateral medial anterior hemimeniscectomy in 18 rabbits (cohort B). Rabbits were allowed to heal for 4 weeks, at which time they were given an injection of experimental therapy (2 × 10^6^ or 6 × 10^6^ AMSCs or lactated Ringer’s) into their right knee and lactated Ringer’s control therapy into the left knee. Rabbits were evaluated for adverse reactions and subsequently sacrificed 4 weeks after experimental injections.

#### Evaluation of Systemic Adverse Reactions

To assess for systemic adverse reactions of AMSC injections into rabbit joints that have undergone meniscectomy, we recorded the weight, body temperature, injection site appearance, and behavior of the rabbits weekly. One rabbit experienced a left knee infection after the bilateral partial medial meniscectomy procedure. This rabbit was sacrificed because of persistent disability at the recommendation of the veterinary staff. The sacrifice was performed before the rabbit received an experimental injection; thus, this adverse event was not related to AMSC therapy. Aside from this single rabbit, all other animals maintained normal behavior and stable vital signs. All the remaining animals in cohort B (*n* = 17) were sacrificed 4 weeks after injection therapy (8 weeks after partial meniscectomy) so that intra‐articular tissues could be evaluated for local adverse reactions.

#### Assessment of Tissue Morphology/Histology in Rabbit Knees After Partial Meniscectomy

**Figure 5:**
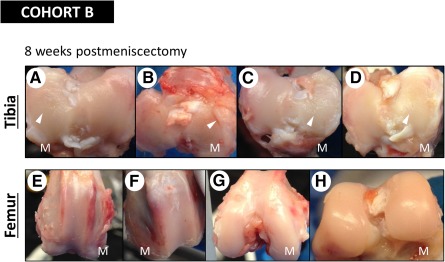
Gross morphology of articular surface after meniscectomy (cohort B). Images of rabbit tibias and femurs extracted 8 weeks after meniscectomy depicting gross morphology. **(A):** Left tibia, lactated Ringer’s. **(B):** Left tibia, 2 × 10^6^ cells. **(C):** Right tibia, lactated Ringer’s. **(D):** Right tibia, 6 × 10^6^ cells. **(E):** Right femur, 6 × 10^6^ cells. **(F):** Left femur, lactated Ringer’s. **(G):** Right femur, 6 × 10^6^ cells. **(H):** Right femur, lactated Ringer’s. Arrowhead, signs of roughness. Abbreviation: M, medial.

The rabbit knees were dissected after sacrifice to assess whether injection of AMSCs into the joint had any morphological or immunological impact on the integrity of the rabbit knee. In cohort B animals, a roughened cartilage surface was evident on the medial articular surface of all tibia specimens across all treatment groups (2 × 10^6^ or 6 × 10^6^ AMSCs or lactated Ringer’s solution) (Fig. 5). These findings are consistent with an early osteoarthritic state in a meniscetomized joint. The surface roughness that was readily identifiable upon visual inspection did not, however, translate into histologically observable measures of osteoarthritic change.

**Figure 6:**
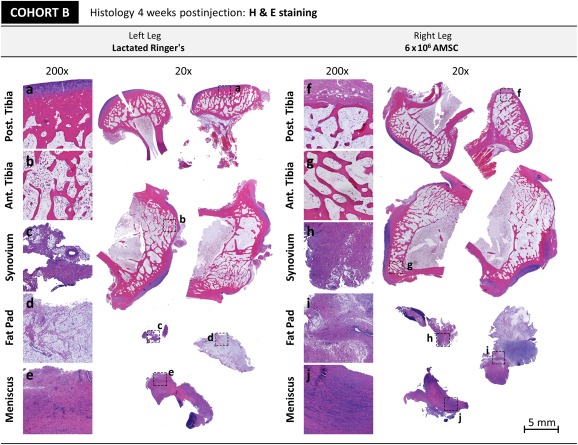
Cohort B histology: 6 × 10^6^ AMSC. H&E‐stained histological samples of cohort B rabbit right knees injected with 6 × 10^6^ AMSCs and the corresponding control left legs (lactated Ringer’s) are shown. Tissues include the tibia, synovium, infrapatellar fat pad, and meniscus. **(a–g):** No significant pathology was observed in left leg tissues **(a–e)** or in the right tibia samples **(f, g)**. **(h–j):** The synovium **(h)**, infrapatellar fat pad **(i)**, and meniscus **(j)** had mild inflammation and aggregation of lymphohistiocytic cells, with signs of mild subintimal edema in **(h)** and **(j)**. Scale bar = 5 mm. Abbreviations: AMSC, adipose‐derived mesenchymal stem cell; Ant.Tibia, anterior tibial tubercle side; H&E, hematoxylin and eosin staining; Post.Tibia, posterior tibial fibular side.

After gross examination, knee tissues, including the tibia, meniscus, synovium, and fat pad, were harvested. Representative histological pictures of control‐ and AMSC‐treated knees are presented in Figure 6 and supplemental online Figures 1–3. Based on the OARSI scoring of the safranin O slides, the grading for evaluable cartilage in all cohort B tibia samples was as follows: 0, uniform safranin O staining; 0, structure normal; 0, no decrease of cells; and 0, no cluster formation. In some samples, focal or multifocal artifactual fissures were observed, where hypocellularity or loss of safranin O staining adjacent to the cleft was present. There were no observable differences in cartilage specimens according to treatment side (left vs. right) or across animal treatment groups. Furthermore, staining provided no evidence of changes or damage to the articular cartilage.

To supplement the OARSI scoring system, a histopathological assessment of OA synoviopathy in the right and left synovium and fat pad tissues of cohort B animals receiving injections of either (a) lactated Ringer’s or (b) 6 × 10^6^ AMSCs in the right leg was performed. An attempt was made to implement the 0–3 scoring criteria for synoviocytes, inflammatory infiltrates, and synovial stroma according to the parameters outlined by Laverty et al. [26]. A general pathological assessment of all H&E‐stained tissues was also conducted. All of the soft tissues evaluated in cohort B received an OARSI score of 0 (normal or absent) for all relevant categories. In a few samples, abnormal histologic findings were also noted and included focal areas of necrosis, foreign body giant cells, acellular debris, and edema, which appeared to be spurious findings unrelated to the treatment that was administered. The right knees in the experimental group that were injected with higher‐dose AMSC therapy showed a greater proportion of plasmalymphocytic reactions, with evidence indicative of inflammation, aggregation of lymphoplasmytic cells, and edema. All of the rabbits that received 6 × 10^6^ AMSCs developed cellular infiltrates in their right joint tissues; 50% of the rabbits receiving 2 × 10^6^ AMSCs developed infiltrates; and only 40% of rabbits receiving lactated Ringer’s solution developed infiltrates. In the left contralateral control knees, we observed very little significant pathology; in isolated cases, mild edema or minimal aggregation of lymphoid cells was observed. In addition, necrotic debris was observed in the fat pad of an animal with lactated Ringer’s and in the fat pad and synovium of two animals injected with 6 × 10^6^ AMSCs. As in cohort A, these studies suggest that human AMSCs may promote cellular infiltration in joint tissues, because rabbits receiving AMSC therapy had a higher proportion of cellular infiltrations upon histologic analysis of tissues. This reaction is consistent with a normal response to xenograft AMSCs in an immunocompetent animal and did not produce a clinically significant systemic reaction or cause measurable damage to intra‐articular joint tissues.

#### Pain Assessment (Incapacitance Testing)

A quantitative measure of the level of discomfort (incapacitance) as it relates to injury or inflammation was collected for each rabbit in cohort B to assess whether the injection of stem cells caused the subjects to preferentially shift their weight to a specific leg secondary to pain [28, 29]. Across all rabbits, incapacitance measures (in grams) for the neutral position (N) are as follows: N_Right_ = 1,151.4 ± 173.9 g and N_Left_ = 1,089.0 ± 229.0 g for the right and left side respectively, with no significant difference between legs (*p* = .3967). Likewise, no significant difference (*p* = .5835) was found between legs in the elevated (E) position: E_Right_ = 1,327.8 ± 324.9 g compared with E_Left_ = 1,280.5 ± 235.6 g (supplemental online Fig. 4). Statistical analysis also revealed no differences due to treatment in the right (*p* = .7992) and left (*p* = .5237) legs or between leg and treatment type (*p* = .9461). The intraclass correlations (with 95% confidence intervals) and coefficients of repeatability for each leg and position are presented in supplemental online Figure 4. These findings indicate no statistically significant weight‐bearing preference in any of the rabbits. Thus, the lymphoplasmacytic reactions related to AMSC therapy did not result in measurable pain behavior in our rabbit model.

#### Radiographic Studies (X‐Ray and Magnetic Resonance Imaging)

Bilateral AP and lateral knee x‐ray radiographs were taken at the time of surgical meniscectomy, knee joint injection, and at the time of final sacrifice to evaluate for any pathologic changes in the knee joint. X‐rays reveal no signs of osteoarthritic change related to AMSC therapy, as assessed by joint space narrowing, sclerosis, osteophyte formation, and joint deformity (supplemental online Fig. 5) stemming from joint destabilization upon partial meniscus removal.

**Figure 7:**
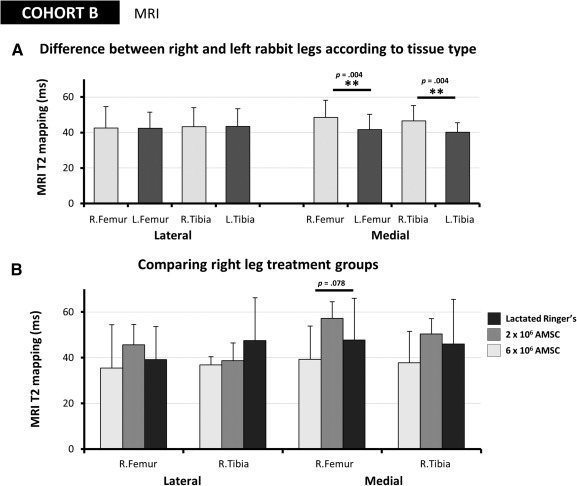
Rabbit magnetic resonance imaging. Magnetic resonance T2‐weighted maps were generated for each rabbit knee, and the average T2 relaxation times in milliseconds for the articular cartilage of the lateral and medial femoral condyles and tibial plateaus were measured. **(A):** No significant differences were detectable between left and right legs for different lateral tissues (lateral femur, *p* = .904; lateral tibia, *p* = .965), whereas the right medial femur and right medial tibia had significantly higher (*p* = .004) T2 times compared with the left sides. **(B):** No significant differences in T2 were observed between lactated Ringer’s or 2 × 10^6^ or 6 × 10^6^ AMSC‐injected right legs (lateral femur, *p* = .792; lateral tibia, *p* = .427; medial femur, *p* = .078; medial tibia, *p* = .743). ∗∗, *p* < .005. Abbreviations: AMSC, adipose‐derived mesenchymal stem cell; L., left; MRI, magnetic resonance imaging; R., right.

T2 mapping by magnetic resonance imaging was performed on disarticulated rabbit legs from cohort B to develop a quantitative description of joint degeneration resulting from bilateral meniscectomies and the injection of AMSCs. Damage to articular cartilage leads to an increase in the amount of free water in the tissue, increasing T2 relaxation times [30, 31, 32]. Higher T2s are therefore reflective of abnormal or damaged cartilage, predicting early degenerative change [33, 34]. Significant increases in T2 times in milliseconds were observed for both the right medial femur (RF_M_ = 48.5 ± 9.7 milliseconds) and right medial tibia (RT_M_ = 46.5 ± 8.8 milliseconds) compared with the left medial femur (LF_M_ = 41.7 ± 8.6 milliseconds) and left medial tibia (LT_M_ = 40.2 ± 5.3 milliseconds), with *p* = .0037 and *p* = .0038, respectively (Fig. 7A). No significant differences, however, were detectable between right and left legs for different lateral tissues types: right lateral femur (RF_L_ = 42.6 ± 12.0 milliseconds) compared with the left lateral femur (LF_L_ = 42.3 ± 9.2 milliseconds), with *p* = .9036, and right lateral tibia (RT_L_ = 43.3 ± 10.7 milliseconds) compared with the left lateral tibia (LT_L_ = 43.4 ± 10.0 milliseconds), with *p* = .9647. T2 mapping was also compared across tissues types in the right leg to test for changes due to treatment. No significant differences in T2 times were observed between lactated Ringer’s, 2 million, or 6 million AMSC‐injected right legs (Fig. 7B). We conclude that, although imaging parameters do not differ for lateral tissues, partial removal of the medial meniscus likely accounts for T2 differences observed in the medial articular cartilage.

## Discussion

Current treatments for OA are not able to restore normal articular cartilage integrity [3]. Because of their differentiation capacity, as well as anti‐inflammatory and regenerative trophic functions, stem cells represent a novel treatment option for OA that warrants further investigation in human clinical trials [35, 36, 37, 38]. Our focus on AMSCs as a resource is primarily driven by their ease of isolation in large quantities as a clinical‐grade product that is expanded in human platelet lysate and used in autologous cell transplantation [11]. Potential risks related to the use of stem cells include colonization of nontarget tissues, possible induction or stimulation of tumorigenesis, and contamination and lack of universal protocols during isolation, as well as possible inadvertent biological complications that may arise during cell expansion [39]. The present study was designed to assess the safety and reliability of platelet‐expanded human AMSCs for possible use in clinical trials to both relieve pain symptoms and/or mitigate the progression of early stages of OA, especially in patients with acute joint injuries (e.g., meniscus tears). We examined the safety of human AMSCs after injection using two different protocols. In the first cohort (cohort A), cells were delivered into healthy rabbit knees by minimally invasive intra‐articular injection, with the primary goal of evaluating for any acute adverse reactions. In the second cohort, we evaluated the response of the joint to AMSC injection 4 weeks after performing a partial anterior hemimeniscectomy. This cohort was meant to simulate a clinical scenario in which a patient sustains a meniscal injury and is predisposed to early osteoarthritis development. Future studies examining AMSC treatments at both earlier and later time points may reveal different degrees of efficacy under different clinical scenarios.

We did not observe serious adverse reactions (death, systemic reactions, or tissue damage) upon intra‐articular injection when healthy rabbits maintaining normal biometric parameters received this experimental AMSC treatment. Histologic evaluation of tissues did reveal a higher incidence of cellular infiltration in knees receiving AMSC therapy. The latter response has been observed in previous animal models of OA and/or MSC studies [40, 41, 42], but it did not translate into evidence of major structural damage to cartilage or other intrasynovial tissues, as evident from the OARSI scoring for articular cartilage or synoviopathy.

Previous studies have shown that transplantation of isolated human amniotic mesenchymal cells [43], human bone marrow stromal cells (BMSCs) [40, 44], and others [42] into rabbit knee joints does not elicit apparent inflammatory responses. However, in our study we observed lymphoplasmacytic infiltration and inflammation in both cohorts. Our findings are consistent with other studies showing immune responses related to xenogeneic transplantation [42] and nonautologous transplantation of isolated cells [40, 45]. It appears there is no consensus on the biological effects of heterologous cell transplantation in rabbits.

Although OA‐related joint degeneration can be mimicked in the rabbit via chemical induction, anterior cruciate ligament transection (ACLT), or meniscectomy [46, 47, 48, 49], our studies applied the latter procedure to assess the safety of AMSCs in this more clinically relevant environment. No adverse reactions related to AMSC use were observed in this cohort, except for one animal that was removed from our study before stem cell injection due to development of a left knee infection after meniscectomy. Pain outcomes related to AMSC injections were evaluated in all animals by testing incapacitance as described in several recent studies [49, 50, 51]. We did not observe significant weight‐bearing asymmetry between treatment and control groups 4 weeks after AMSC injections in cohort B, nor did we observe changes in spontaneous locomotor activities or gait, suggesting that cell treatment neither contributes to nor alleviates pain in the rabbit hind limbs at this time point. In this study, we limited incapacitance testing to terminal time points just before euthanasia to avoid stressing the rabbits, because the rabbits often became very agitated during handling. Future assessments of knee pain with incapacitance testing using more frequent, as well as earlier and later, time points may reveal significant pain‐related findings.

As anticipated, gross morphological inspection of the joints after sacrifice suggested early signs of cartilage dysregulation, evidenced by the presence of mild fibrillation of the articular cartilage. Radiological examination of these same joints did not reveal major areas of joint space narrowing, sclerosis, or osteophyte formation, and we did not find indications for structural cartilage degeneration by H&E staining or safranin O‐fast green staining. Hence, the meniscectomy procedure carried out in our study generated mild preclinical OA symptoms that were far less pronounced than the radiological and/or histological signs of OA that have been previously reported at 8–24 weeks after surgery in models of ACLT [17, 36, 52] and meniscal injury [35, 53, 54].

This safety study also provides additional preliminary information regarding cartilage damage by measurements of MR relaxation time T2. Degeneration changes have been shown to increase T2 times in human articular cartilage [55], particularly when comparing healthy and OA‐damaged subjects [56]. Although we did observe increases in T2 in the right medial knee tissues compared with their left counterparts, no significant differences were found across treatment types, suggesting that any measureable changes in cartilage structure were not due to AMSC dose alone. Because T2 signals are highly sensitive to various factors (e.g., background, orientation) and there is the potential for substantial heterogeneity within healthy cartilage, interpretation of these results is challenging [57]. Additional studies are currently ongoing to fully realize the clinical values of these types of radiographic parameters.

Our findings indicate that, aside from the presence of cellular infiltrates in the rabbit joint tissues, there is minimal risk associated with AMSC injection into the animals at the doses we used (2 × 10^6^ to 12 × 10^6^). This study contributes to the growing evidence that MSCs can be used safely in vivo for the duration of our experiments (up to 4 weeks). In our study, we focused on relatively short time points (2–4 weeks) to ensure that we would be able to capture acute adverse reactions directly related to the AMSC therapy. However, studies examining longer time points after AMSC injections, as well as multiple dosing regimens with autologous cell therapies, would be interesting, particularly given that recent studies have shown that MSCs may have the potential to persist within the joint space and joint tissues for longer periods of time after intra‐articular administration [58]. A number of preclinical studies evaluating the regenerative potential of BMSCs, AMSCs, and other types of MSCs after meniscus injury provide evidence of tissue regeneration, protection against joint surface irregularities, and improvement in cartilage quality over controls [35, 36, 53, 54]. This response is potentially because of trophic mechanisms of action, via the release of growth factors and cytokines, MSC adherence and differentiation, and extracellular matrix deposition [17, 18], although more rigorous studies on the influence of catabolic and inflammatory environment during OA are necessary to elucidate the specific mechanisms involved. The safety and efficacy of intra‐articular injections of bone marrow‐derived MSCs has been evaluated in patients with knee OA [59, 60, 61], with no measured adverse events and reports of pain reduction and improved function. In a recent publication, Vangsness et al. [18] followed patients in a phase I clinical study for 2 years, with good demonstration of MSC safety and relative improvements in pain. It remains unclear, however, whether clinically relevant increases in meniscal regeneration are possible. Improvements in areas such as dose optimization, standardization of outcome measures, evaluation of the full joint environment, stem cell tracking and survival, and the feasibility of repeat injections will need to be explored further to balance OA treatment with the safe delivery of MSCs.

Although our results suggest that human AMSC injections are well tolerated in the rabbit model, a number of limitations still need to be addressed. Although the rabbit is a practical model for early stages of evaluation because of cost‐effectiveness, handling, and reasonable joint size [62], rabbits are associated with spontaneous intrinsic healing of cartilage—a property generally not seen in humans [46, 52, 63, 64]—and have distinctly reduced meniscus vascularization (compared with humans) [65], which could significantly alter the disease environment and influence outcomes. A longer follow‐up period, with the potential to track cells after injection, could provide more detailed information about cell fate, cell impact, and OA induction. An area of ongoing investigation in the field of translational medicine is whether human cells that will ultimately be administered to patients or cells derived from the animal model under study should be used for safety investigations. In this study, a safety evaluation of human‐derived cells applied to a rabbit model of OA proved sufficient for regulatory approval in the setting of prior experience with MSC therapy. However, there is still a strong need for future investigations to explore the differences between MSCs across species to understand what factors are important for safety and efficacy evaluations and to determine which aspects can help optimize cell‐based manufacturing practices. In our studies, we observed immune reactions associated with xenogeneic AMSC therapy. Previous experiences with the use of autologous AMSC therapies in patients, for the treatment of diseases outside of osteoarthritis, have not shown evidence of adverse immune reactions [66, 67]. However, without these prior human studies, many of which have been for diseases associated with high morbidity that lack effective therapies, the translational barriers for more benign diseases such as OA can be challenging to overcome, given the state of current translational models for cell‐based therapies.

## Conclusion

We present the results of a study evaluating the safety of human adipose‐derived mesenchymal stromal/stem cells delivered as single intra‐articular injections (at three dose levels) into normal healthy rabbit knees and into knees at risk for the development of osteoarthritis after bilateral medial meniscectomy. Overall, study injections were well tolerated up to 4 weeks after injection, demonstrating no major adverse effects on tests of general health, function and behavior of animals. Likewise, we did not find imaging (radiographic or MR), gross anatomic, or histologic evidence postmortem of concerning risk in this study. The results suggest that concentrated human AMSCs can be safely delivered to the rabbit knee joint. Our in vivo studies should contribute to the use of MSC‐based therapeutics in phase I clinical trials in patients with OA.

## Author Contributions

S.M.R.: conception and design, collection and/or assembly of data, data analysis and interpretation, manuscript writing, final approval of manuscript; J.M.D., D.L.J., and J.S.: collection and/or assembly of data, data analysis and interpretation, manuscript writing, final approval of manuscript; Y.L. and M.A.F.: collection and/or assembly of data, data analysis and interpretation, final approval of manuscript; T.d.M., E.A.L., H.N., C.R.P., D.J.R., A.D., and E.T.C.: collection and/or assembly of data, final approval of manuscript; D.R.L., W.Q., A.J.K., and H.‐J.I.: data analysis and interpretation, final approval of manuscript; A.B.D.: provision of study material or patients, data analysis and interpretation, final approval of manuscript; A.J.v.W.: conception and design, financial support, data analysis and interpretation, manuscript writing, final approval of manuscript.

## Disclosure of Potential Conflicts of Interest

A.B.D. is on the board of directors and has stock options with Mill Creek Life Sciences and has rights to royalties. J.S. is an employee of Sonex Health, LLC, and has intellectual property rights and stock options with Tenex Health, Inc., and Sonex Health, LLC. The other authors indicated no potential conflicts of interest.

## Supporting information

Supporting Information
